# Hybrid Feature Extractor Using Discrete Wavelet Transform and Histogram of Oriented Gradient on Convolutional-Neural-Network-Based Palm Vein Recognition

**DOI:** 10.3390/s24020341

**Published:** 2024-01-06

**Authors:** Meirista Wulandari, Rifai Chai, Basari Basari, Dadang Gunawan

**Affiliations:** 1 Department of Electrical Engineering, Universitas Indonesia, Depok 16424, Jawa Barat, Indonesia; meirista.wulandari@ui.ac.id (M.W.); basyarie@eng.ui.ac.id (B.B.); 2 School of Science, Computing and Engineering Technologies, Swinburne University of Technology, Hawthorn, VIC 3122, Australia; rchai@swin.edu.au; 3 Research Center for Biomedical Engineering, Faculty of Engineering, Universitas Indonesia, Depok 16424, Jawa Barat, Indonesia

**Keywords:** CNN, DWT, HOG, palm vein, VeinCNN

## Abstract

Biometric recognition techniques have become more developed recently, especially in security and attendance systems. Biometrics are features attached to the human body that are considered safer and more reliable since they are difficult to imitate or lose. One of the popular biometrics considered in research is palm veins. They are an intrinsic biometric located under the human skin, so they have several advantages when developing verification systems. However, palm vein images obtained based on infrared spectra have several disadvantages, such as nonuniform illumination and low contrast. This study, based on a convolutional neural network (CNN), was conducted on five public datasets from CASIA, Vera, Tongji, PolyU, and PUT, with three parameters: accuracy, AUC, and EER. Our proposed VeinCNN recognition method, called verification scheme with VeinCNN, uses hybrid feature extraction from a discrete wavelet transform (DWT) and histogram of oriented gradient (HOG). It shows promising results in terms of accuracy, AUC, and EER values, especially in the total parameter values. The best result was obtained for the CASIA dataset with 99.85% accuracy, 99.80% AUC, and 0.0083 EER.

## 1. Introduction

Biometric measurement has been increasingly used for human recognition, such as in system security for authentication systems [1]. Biometrics is a combination of science and technology that measures biofeatures, such as behavioral or physical, to identify an individual [2]. Since they are attached to the human body, biofeatures are considered to be more effective and robust than conventional forms of identity, such as personal identification numbers (PINs), passwords, or cards [3], which are susceptible to being forgotten or stolen [4]. Based on their visibility, biofeatures can be divided into extrinsic and intrinsic biometrics based on their characteristics, each with their advantages and disadvantages. Extrinsic biometrics are visible, while intrinsic biometrics are perceived indirectly. As the most widely used extrinsic biometric, fingerprint recognition is durable and consistent but still faces issues related to image distortion, the clarity of the core of the fingerprint, and insufficient data [5,6]. Among other types of extrinsic biometrics, face recognition relies on the use of a picture and coordinates, and external conditions such as illumination, occlusion, and the person undergoing some physical changes can lead to unreliable recognition [7]. The iris is a thin and secure organ that is least affected by external conditions, but the durability of iris recognition is sensitive to eye clarity, pupil size changes, radiation, illumination, and the alignment of the camera [8]. On the other hand, intrinsic biometrics are more advanced in terms of data acquisition than extrinsic biometrics. Their location inside the body makes these biometrics difficult to forge and imitate, which can avoid fraud [9]. As a result, some advanced methods or tools are needed to acquire and view hidden biometrics, using medical equipment such as electrocardiogram signals, magnetic resonance imaging (MRI), X-ray, and infrared spectroscopy [10,11]. The high cost of acquiring MRI or X-ray images is a consideration of this biometric system [12]. The infrared spectrum can penetrate the human skin, and it is absorbed by hemoglobin and other vascular structures. The different amounts of absorption show the vein pattern in a captured image [13]. Among the different types of intrinsic biometrics, palm veins are one of the most promising. The vein pattern is unique from one human to another. In addition, the advantages of the use of palm veins as a biometric are that it is consistent [14] and the veins only exist in a human who is alive [15,16]; it is also accurate, contactless, cost-effective, convenient, and reliable [17,18]. The veins carry blood containing hemoglobin, which can be captured using an infrared camera with a wavelength spectrum of 750 nm–1 mm [19].

However, the vein pattern is difficult to capture due to some factors causing the infrared light that penetrates the human skin to be imperfectly absorbed by deoxygenated hemoglobin [20], such as scars and hair on the skin [21]. There are several steps to obtain good recognition of the palm veins, including palm vein acquisition, image preprocessing, feature extraction, and classification. Image acquisition can be achieved using the infrared spectrum for palm vein acquisition. The vein image should be filtered during the preprocessing of the entire recognition system. In addition to the appearance of noise, other problems in infrared images are irregular texture and low contrast [22,23]. When using appropriate devices, palm veins can be combined with a palm print to produce images that can be processed [24]. Still, since palm vein images are irregular and noisy, more images must be obtained of the palm veins themselves to lead to better recognition.

The image filter methods commonly used for image preprocessing include the Laplacian filter [25], the Gaussian filter [26], the hybrid Wiener and median filter [27], the Canny detector and Gabor filter [28], and discrete wavelet transform (DWT) [29]. Among others, the DWT method is superior for image processing since it has image segmentation and denoising characteristics [30]. It transforms and decomposes an image into four sub-band images: coefficient approximation, coefficient detail in horizontal, coefficient detail in vertical, and coefficient detail in diagonal. The approximation detail can describe the information in an image.

The process then continues to feature extraction, which can be performed based on two categories of low-level features, namely, structural and textural features. There are several methods for this category of features, such as supervised discriminative sparse principal component analysis neighborhood-preserving embedding (SDSPCA-NPE) [31], local binary pattern (LBP) [32], gray-level co-occurrence matrix (GLCM), and histogram of oriented gradient (HOG) [33]. Based on the results of [33], HOG showed the best result among the texture features that were extracted [33] due to its superiority in detecting the degree of differences among transformations and variants [34], although there are still some reports that low-level features are unrepresentative and unstable [35]. In order to handle this, methods to extract high-level features have been introduced, i.e., deep learning, and can obtain features automatically from a given dataset for each specific application. This method is promising and has been developed using numerous methods, such as deep neural network (DNN), deep belief network (DBN), and convolutional neural network (CNN). Many researchers have expanded biometric classification using the CNN method to obtain more appropriate image recognition. Some biometrics, such as handwriting [36] and finger veins [37], have been researched using CNN with better pattern recognition accuracy [38].

Since palm veins are irregular and undoubtedly unique, using more images will result in better recognition. The use of CNN has been investigated by many researchers for vein recognition, such as finger veins [39,40], hand dorsal veins [41,42], and palm veins [43,44]. Hong et al. [45] developed a CNN for finger vein recognition using three different datasets based on the quality of the images: good, middle, and poor quality. There are three structures of deep learning to be examined: AlexNet, VGG16, and VGG19. VGG16 with fine-tuning obtained the best result among those three methods. Wang et al. developed a system to recognize two contactless palm vein datasets using a Gabor filter as a feature extractor and a lightweight CNN [46]. Wan et al. [39] examined palm dorsal vein images in three datasets. There are various numbers of subjects and images in each dataset. The three datasets have the same image sizes, but they consist of different quality images. The first and third datasets have better quality images than the second dataset. VGG19 obtained a good result, with an accuracy of 99.7%.

Other researchers of vein recognition have proposed various methods using modified CNN structures to overcome the issue of low discriminative ability in palm vein pattern recognition. Wang et al. [47] modified pretrained VGG-16 with spatial pyramid pooling to obtain discriminative features and achieved an EER of 0.068%. Wang et al. in [48] developed VGG16 with minutiae feature kernel to obtain a better result for EER. Another hybrid deep learning method was introduced by Hou and Yan [49]. The developed hybrid deep learning method with a CNN autoencoder was used to recognize finger veins. The input images were processed by the autoencoder to obtain the discriminative features, and the structure of the CNN was simpler. However, very few research reports provide classification analyses on several different datasets. Therefore, this paper proposes a palm vein recognition method based on CNN, DWT, and HOG for hybrid feature extraction and several datasets so that this feature extractor can be used in more general conditions. The datasets in [50,51,52,53,54] are well-known contact and contactless palm vein image datasets, and are often used to show the state of the art in method improvements, as provided in Table 1.

The proposed DWT and HOG hybrid feature extractor is expected to produce an efficient and effective method with a small error rate value in palm vein recognition. The DWT assists in filtering the noise in the original images, and the HOG improves the sensitivity of the gradient magnitude and gradient orientation of the palm vein image information based on image texture. The texture image part, which contains directional information, has a higher value than the nontexture image part, which does not contain directional information. This filtered image is then prepared for the advanced step that contains only the essential information. In this paper, the evaluation of the recognition system is based on several key performance indicators: the accuracy value, area under receiver operating characteristic curve (AUC), and equal error rate (EER).

From the aforementioned research results, we propose a method to recognize palm vein feature biometrics. The key contributions of this paper are as follows:

- A simple CNN hybrid structure with a feature extraction method to verify the palm vein pattern based on an image. Using the hybrid DWT and HOG as the feature extractor will handle the irregularity and unique properties of the images.
- The proposed hybrid DWT-HOG VeinCNN is implemented in five datasets of palm vein images in one study to understand the general condition of palm vein images.
- The proposed CNN structure can maintain satisfactory accuracy while minimizing the equal error rate.

This research paper’s outline is organized as follows: Section 2 shows the methodology, including image acquisition, preprocessing, feature extraction, classification, evaluation, and error metrics. Section 3 shows the results of the paper. Section 4 presents the discussion. The conclusion is shown in Section 5.

## 2. Materials and Methods

**Figure 1:**
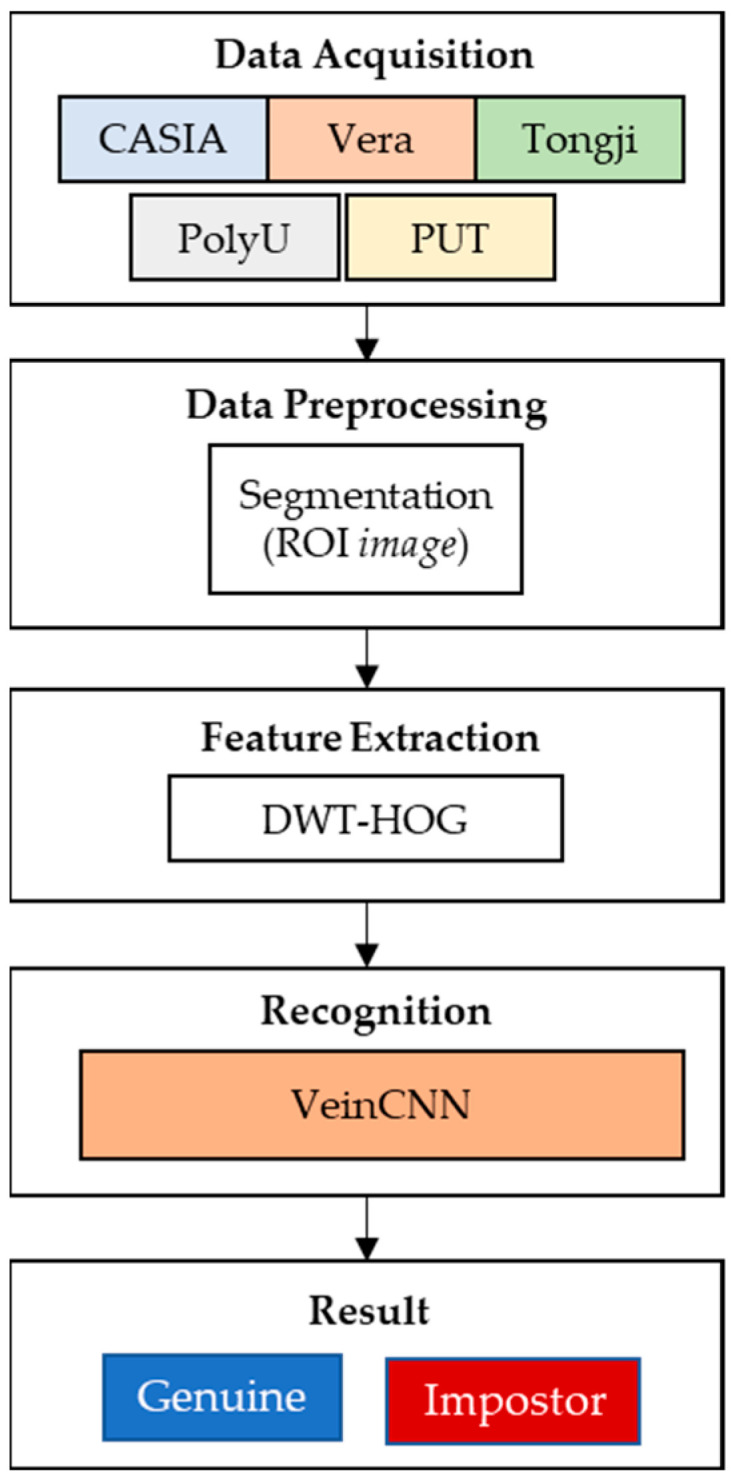
Palm vein recognition with hybrid DWT-HOG VeinCNN.

The method consists of five steps for palm vein recognition, as shown in Figure 1. Step 1 is data acquisition, where images are obtained from five datasets [50,51,52,53,54]. In step 2, each image from the dataset is segmented by applying the preprocessing data to obtain only the part of the image that contains the most information, i.e., the ROI.

An image of the center of the palm is obtained from the entire hand image. This partial image is processed through feature extraction in step 3. In step 3, the images are processed by convolutional mathematics to obtain some features based on DWT and HOG. In step 4, the feature recognition process is performed. The output classification is given in step 5. The result shows whether the image could be genuine or an impostor.

### 2.1. Image Acquisition

**Figure 2:**
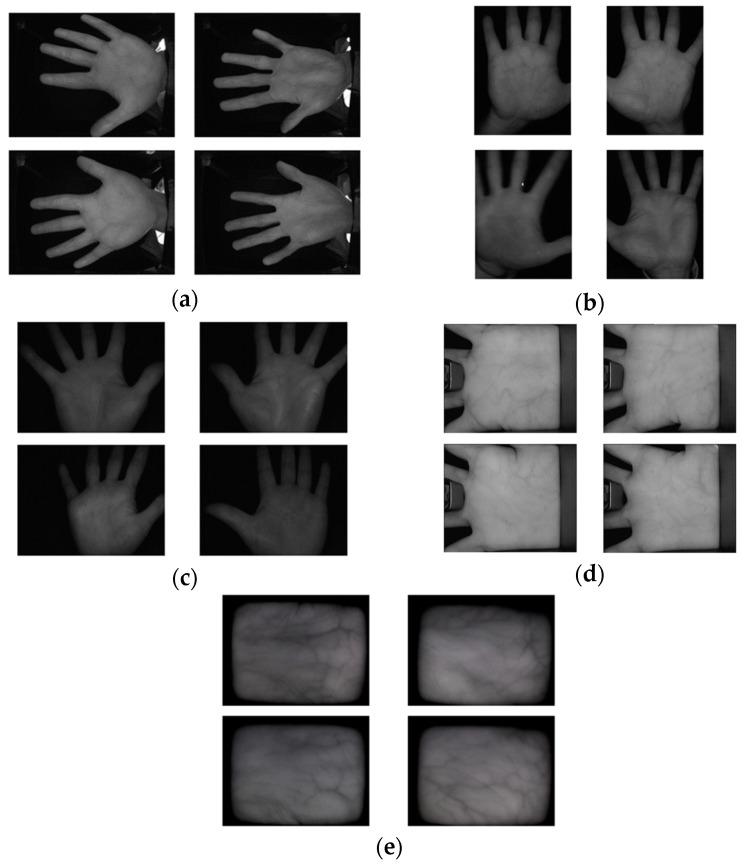
Examples from palm vein datasets: (**a**) CASIA, (**b**) Vera, (**c**) Tongji, (**d**) PolyU, and (**e**) PUT.

This palm vein recognition technique based on images with CNN, DWT, and HOG is proposed because of the great accuracy published in [57,58]. This method is evaluated using five public datasets available online: CASIA, Vera, Tongji, PolyU, and PUT datasets. These public datasets assist researchers in analyzing the proposed algorithm and comparing it with previous algorithms, and the possibility of permission being granted for research or academic purposes is high. An example of each dataset is shown in Figure 2. The details of each dataset are summarized in Table 2.

#### 2.1.1. CASIA

This contactless dataset was obtained and captured by the Chinese Academy of Sciences Institute of Automation (CASIA) [50]. It consists of 100 subjects using their hands, left and right, which are considered as two different individuals. Each palm was captured six times in two sessions with six infrared spectra (460, 630, 700, 850, 940 nm and white light spectra). The images are represented as an 8-bit grayscale image. There are a total of 7200 palm vein images in this dataset in JPEG image format. The resolution of the images is 768 × 576. However, this paper used only 850 and 940 nm infrared spectrum images to obtain a clearer pattern of palm veins and achieve a lower error rate.

#### 2.1.2. Vera

This dataset was collected using a contactless sensor by the University of Applied Sciences Western Switzerland and the Idiap Research Institute [54]. Palm vein images were captured with a wavelength spectrum of 940 nm. There are 110 participants with left and right hands in this study. Each hand was captured five times in two sessions. As a result, the dataset contains 2200 palm vein images in PNG format with an image resolution of 480 × 680.

#### 2.1.3. Tongji

This dataset was collected by Zhang et al. [53] using a contactless sensor for palm veins. The sensor has an infrared camera lens with 940 nm wavelength. Using this spectrum, images of 300 people were obtained. Each palm of an individual was captured 10 times in two sessions. There are a total 12,000 palm vein images in this dataset in BMP image format, and the resolution of the images is 800 × 600.

#### 2.1.4. PolyU

The PolyU dataset was developed by the Hong Kong Polytechnic University [51]. It consists of 250 volunteers with two sessions of imaging. Six images for each palm were captured in each session. In total, the PolyU dataset has 24,000 palm images. It used 880 nm LED infrared. The image size is 352 × 288, and the images are in JPG format (*.jpg).

#### 2.1.5. PUT

The PUT dataset consists of 100 volunteers, with three sessions and 4 images captured in each session [52]. In total, the PUT dataset has 1200 images. It used 880 nm infrared. The image size is 768 × 1024 in BMP format.

### 2.2. Preprocessing Data

**Figure 3:**
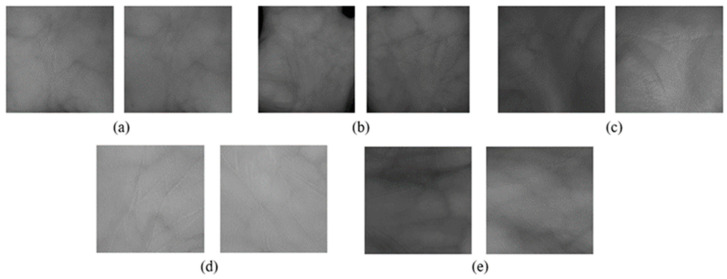
ROI results for (**a**) CASIA, (**b**) Vera, (**c**) Tongji, (**d**) PolyU, and (**e**) PUT datasets.

The veins are captured with a contactless sensor in various infrared spectrum wavelengths and different image formats. The preprocessing step consists of a grayscale image with a further segmentation process. The purpose of the segmentation is to obtain the interest area, generally located in the center of the hand image. Finger valley and hand contour detection methods are applied to obtain the point of reference. In order to overcome issues of contactless sensing such as translation, rotation, and variation in scaling the image, the ROI of the valley between the fingers and centroid in [59] has been applied. The ROI images are resized into 128×128 pixels, and the format of the image is updated to BMP. The input images are normalized. The ROI results of the dataset are shown in Figure 3.

### 2.3. Feature Extraction

Feature extraction is a process of obtaining distinctive features that contain unique information about an object. The features could be extracted by calculating the pixels in a certain area or direction. The feature extraction process plays an important role in palm vein recognition to distinguish an object from others.

There are various approaches to extracting the features of palm veins, such as line, code, and texture features. Texture features are one of the most popular in research. Texture feature extraction involves obtaining features based on the grayscale pixel in the palm vein image and calculating the pixel value or descriptor palm vein image value. The texture feature can provide unique information that distinguishes one palm vein pattern from another. Some of the texture feature methods that are effective for obtaining distinctive features are wavelet feature extraction and histogram of oriented gradient.

#### 2.3.1. Wavelet Feature Extraction

**Figure 4:**
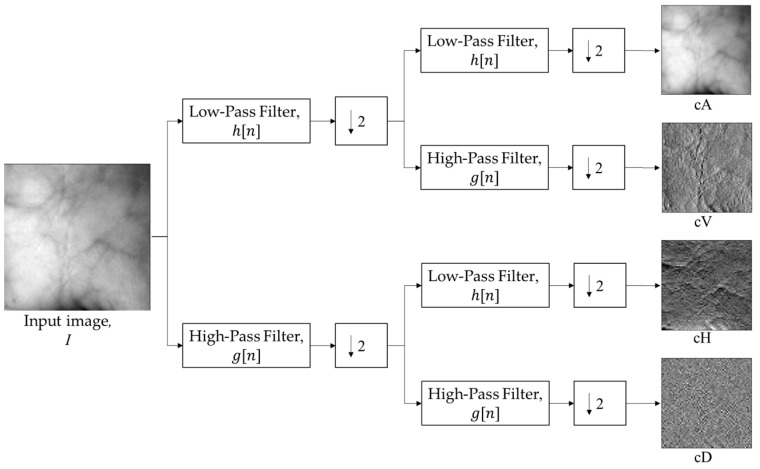
Wavelet feature extraction process.

A wavelet feature is a texture feature that is extracted based on wavelet transformation. Wavelet transformation is applied to the palm vein image to obtain four component images. The components consist of approximation images (cA), a horizontal detail image (cH), a vertical detail image (cV), and a diagonal detail image (cD) [60]. If a palm vein image is symbolized as I, the wavelet transformation generates I = {cA, cH, cV, cD}. The extraction of wavelet features is shown in Figure 4. This approximation image is a result of two low-pass filter processes in wavelet transformation. By using the approximation image only, the classification accuracy result can be increased [61].

#### 2.3.2. Histogram of Oriented Gradient Feature Extraction

Based on [62], a HOG feature is a descriptor feature that has been widely used in image processing. The HOG feature mostly improves the accuracy and quality of the recognition process. The increased accuracy is obtained by utilizing a block, i.e., the smallest gradient on an image. Based on the calculated gradient, the HOG feature has some advantages in terms of the robustness of intensity and direction invariance. The area intensity of the block is then normalized by processing the local histogram value with the remaining cells of the assigned block. This normalization process produces better results in lit and shaded conditions. The vertical and horizontal directions of the gradients are calculated mathematically between the pixel on the image and a certainty kernel factor. The vertical direction gradient represents image direction, while the horizontal direction gradient represents image magnitude. Equation (1) shows the calculation gradient |*G*| of the magnitude based on the intensity of the vertical and horizontal pixels, *I_x_* and *I_y_*. *I_x_* and *I_y_* can be calculated by (2) and (3). On the other hand, the value of the direction image, *θ*, can be found by (4).

(1) $$\left|G\right|=\sqrt{I_{x}^{2}+I_{y}^{2}}$$

(2) $$I_{x}=I*D_{x};\;D_{x}=\left[\begin{array}{ccc} -1 & 0 & 1 \end{array}\right]$$

(3) $$I_{y}=I*D_{y};\;D_{y}=\left[\begin{array}{c} -1\\ 0\\ 1 \end{array}\right]$$

(4) $$\theta =\arctan\left(\frac{I_{y}}{I_{x}}\right)$$

**Figure 5:**
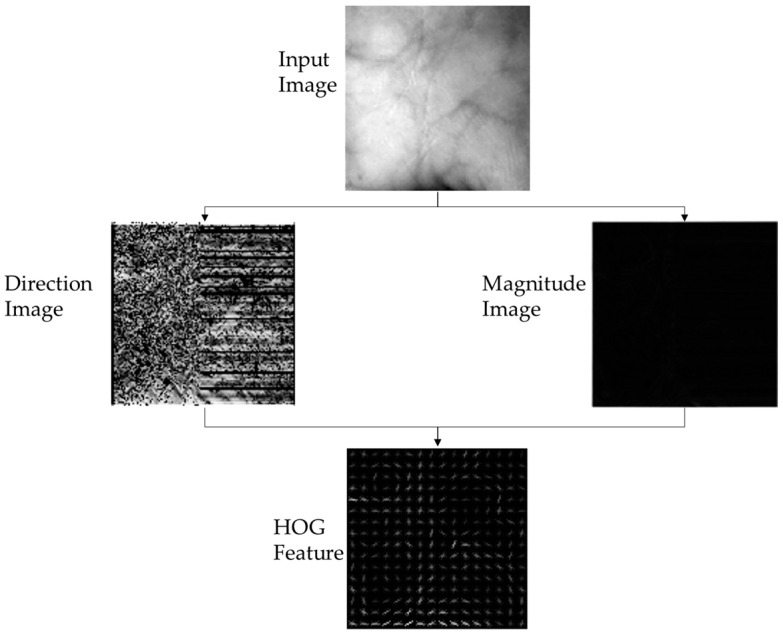
Histogram of oriented gradient feature extraction.

These image direction and image magnitude components are then divided into certain blocks to generate a bin of the histogram structure directions. The arrangement of the bin then produces HOG features, as shown in Figure 5.

#### 2.3.3. Hybrid DWT and HOG Feature Extraction

**Figure 6:**
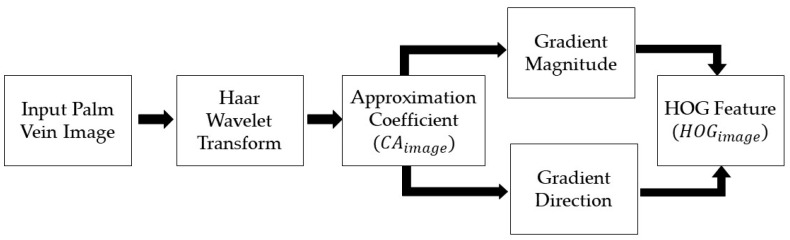
Block diagram of hybrid DWT and HOG feature extraction.

**Figure 7:**
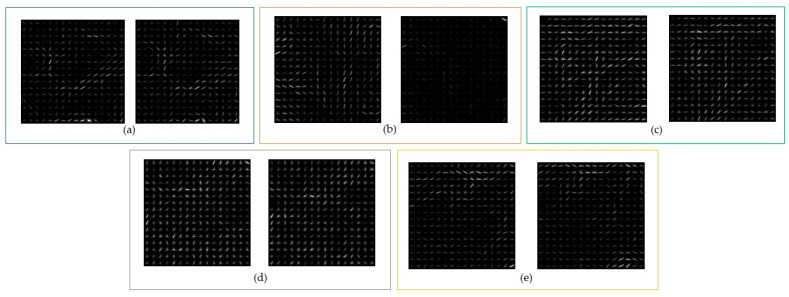
Wavelet and HOG feature extraction for each dataset: (**a**) CASIA, (**b**) Vera, (**c**) Tongji, (**d**) PolyU, and (**e**) PUT.

Combining a wavelet-transformed palm vein image with HOG features shows the essential information of a palm vein image. A block diagram of the hybrid wavelet and HOG feature extraction is shown in Figure 6. The input palm vein image is transformed by Haar wavelet to obtain an approximation coefficient. This approximated image is then calculated to acquire gradient magnitude and gradient direction. Based on these gradients, the HOG feature can be aligned. The procedure of the proposed hybrid wavelet and HOG feature extraction method is shown in Table 3. The results of the proposed method are provided as HOG features in Figure 7a–e for the CASIA, Vera, Tongji, PolyU, and PUT datasets, respectively.

### 2.4. Recognition Based on Convolutional Neural Network

The simulation of palm vein images involves a recognition process that consists of two main processes: enrollment and recognition [63]. The recognition process is carried out to match certain features that are registered and stored in template storage so that the decision module can provide a final decision as to whether or not a person is registered in the database (genuine or impostor) using CNN.

The CNN process includes training and evaluation processes. For the training and evaluation of our proposed method, palm vein images from five datasets containing various numbers of images are used. The images from each dataset were divided into three subsets for training, validation, and testing of 70%, 20%, and 10%, respectively. The simulation was conducted using the Python programming language and the Jupyter Notebook as an integrated development environment (IDE) on the proposed VeinCNN model. Additionally, we adopted the TensorFlow backend with the Keras framework [64]. The parameters of the proposed VeinCNN method were determined to govern the architecture of the network, which were the type and depth of layer, activation function, output shape, kernel size, and number of filters. The layers consist of a convolutional layer, a max-pooling layer, a flattened layer, and a dense layer. To handle the nonlinearity of palm vein images and reduce computational resources, ReLU and sigmoid were used. Since palm vein images consist of detailed lines and edges, small kernel sizes of two and three are more suitable for this simulation. The number of filters was set to 32 and 64 as a power of two so that the simulation process could effectively filter the palm vein images. This proposed method compiled binary cross-entropy as the loss function and root mean square propagation as the optimizer. Early stopping was applied to increase the training efficiency and minimize overfitting.

#### 2.4.1. VeinCNN

**Figure 8:**
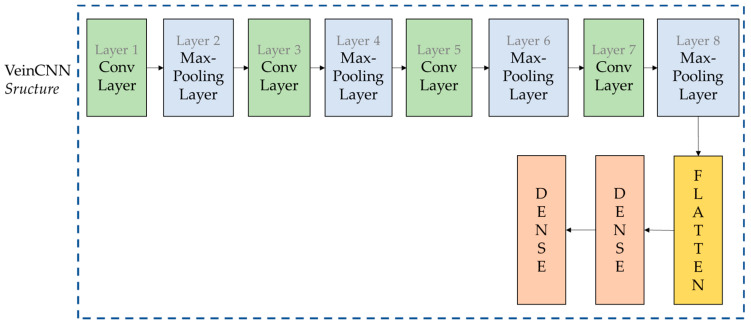
The structure of VeinCNN.

CNN is one of several deep learning methods used to recognize images. However, the arrangement of the CNN must be a consideration to obtain a satisfactory performance, which involves the amount of input data and the network structure. A lack of data will lead to overfitting. Furthermore, the layers in the CNN will also affect the performance. In general, the convolution layer is the first, while the fully connected layer is the last. Convolution layers support the CNN to extract the characteristic features and information of an image. The fully connected layer will decide the number of parameters processed at the end of the network. Restricting the network to a maximum number of parameters should be a consideration, since too many parameters will lead to issues related to an increase in computational resources and overfitting [65]. There are several ways to find an agreement between the amount of data and the number of parameters that should be considered in a CNN for palm vein images. The convolution and max-pooling layers must be set into an optimum configuration in palm vein recognition. Therefore, a CNN configuration is developed in this research to verify the palm vein image, called VeinCNN. The developed VeinCNN applies one input layer, four convolution layers, four max-pooling layers, one flattened layer, and two dense layers, as shown in Figure 8.

The input layer is used to gain a palm vein image that is converted into a 128 × 128 size input with three layers. The next layers are the three convolution layers and four max-pooling layers with kernel size 3 and kernel size 2, respectively. The next step is proceeded by the flattened layer and dense layer. A summary of the network structure of VeinCNN is provided in Table 4.

#### 2.4.2. Vein Recognition Using Hybrid DWT-HOG VeinCNN Feature Extraction

The noise in unclear images of veins can randomly affect the acquisition process and interfere with the recognition process. Filtration of palm vein images using the wavelet transformation method could handle this interference and provide clearer images from which gradient features can be extracted by applying HOG feature extraction. Using an orientation of 9, 8 × 8 pixels per cell and 2 × 2 cells per block, optimum images could be obtained for detecting the vein pattern related to orientation and direction. HOG is capable of gaining information features that are more noise resistant and representative. Detailed and complicated palm vein images can potentially be verified automatically using noise-resistant feature extraction and a CNN network with a more compact structure.

**Figure 9:**
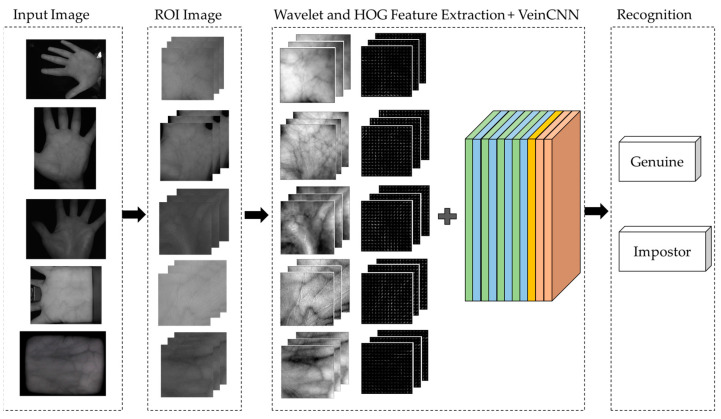
Block diagram of the proposed VeinCNN and wavelet feature extraction HOG combination palm vein recognition process.

The proposed hybrid wavelet and HOG feature extraction method based on VeinCNN is provided by a block diagram in Figure 9, where five palm vein datasets from CASIA, Vera, Tongji, PolyU, and PUT are used, as shown in Table 2. Since this research was conducted on numerous datasets, each dataset is represented by an image in each process in order to provide a simple block diagram. This block diagram represents the performance of palm vein recognition, which is clearly shown by the third step in which the hybridization process is performed.

### 2.5. Performance Biometric Evaluation

An assessment of the biometric system’s performance was conducted. Accuracy, receiver operating characteristics (ROCs), and equal error rate (EER) are frequently used to evaluate performance. Accuracy is a measurement of the reliability of a biometric system. It compares the total true positives and true negatives with the overall total in a biometric system. The ROC curve is a two-axis representation. Both the true-positive rate and the false-positive rate are included in it. There is an area beneath the curve formed by the curves of those two categories. This is the area under the curve (AUC) score. There is a range of 0 to 1. EER is the name given to the junction of the ROC’s diagonal line and curve. EER displays the potential for the biometric authentication system to determine if a given probability is mistakenly positive or negative [66].

## 3. Results

In this paper, recognition is conducted using VeinCNN with a combination of wavelet feature extraction and HOG using five specified datasets. The accuracy, EER, and AUC for each dataset represent the recognition results. To investigate the impact of the feature extraction method, the proposed VeinCNN was used to simulate the recognition process without any feature extraction, which is described as the raw data. Then, the simulation-applied wavelet and HOG, respectively, and the hybrid wavelet and HOG feature extractor were applied sequentially to the VeinCNN structure. The sequential process in deep learning tends to preserve more computational resources, such as GPU resources and workload, compared with the parallel process. Table 5 shows a comparison of the results of the feature extractor.

VeinCNN, which is used to process the raw data and is considered the simplest method, provides the benchmark result for comparison with the other feature extractors. The results for the standalone wavelet feature extraction methods in this case show instability. Meanwhile, the HOG feature extraction methods applied to VeinCNN show conflicting results. In general, HOG feature extraction presents better results than wavelet transformation. Remarkably, the best result appeared when the hybrid wavelet and HOG were applied to VeinCNN. The accuracy and AUC increased in some datasets, and the EER decreased in mostly all datasets. The wavelet transform prepared the image, and HOG shows the edge features based on their gradient and magnitude. Table 6 shows a summary of the accuracy, EER, and AUC results for all datasets. The proposed feature extractor results are promising when compared with published works. On the CASIA dataset, the accuracy obtained by this proposed feature extractor is 99.85% compared with 99.25% obtained using HOG alone [56]. On the PUT dataset, the accuracy obtained by this proposed feature extractor is 99.85% compared with 93.92% obtained in a previous work [55].

## 4. Discussion

### 4.1. Total Parameter

The recognition performance results of VeinCNN using a combination of wavelet feature extraction and HOG are conducted and compared with several transfer learning methods as a benchmark in CNN research. In general, the parameters that are used in the proposed method are simpler than three existing transfer learning methods: AlexNet, VGG16, and ResNet50.

The application of the Hybrid DWT-HOG VeinCNN Method significantly impacts the total number of parameters in the CNN. The VeinCNN hybrid wavelet and HOG method requires 388,546 parameters. This number is much lower than in other transfer learning methods. It is 72 times lower than AlexNet, 38 times lower than VGG16, and 61 times lower than ResNet50.

**Figure 10:**
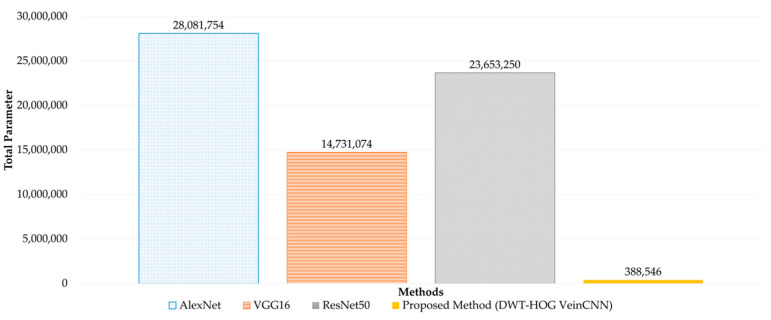
Total parameter in AlexNet, VGG16, ResNet50, and the proposed method.

The slight differences in the accuracy, AUC, and EER values could be compensated for by the lower computational resources. The low number of total parameters means that less computational resources are required. The comparison result of the performance is shown in the points. Figure 10 shows the difference in the total number of parameters in AlexNet [40], VGG16 [65], ResNet50 [66], and the proposed method.

### 4.2. Accuracy

Compared with AlexNet, VGG16, and ResNet50, the proposed recognition scheme Hybrid DWT-HOG VeinCNN is capable of obtaining great accuracy on all datasets, which is shown in Table 7. This proposed scheme gained the highest accuracy of 99.85% and 98.15% on the CASIA and PUT datasets, respectively, and gained the lowest accuracy of 85.97% on the PolyU dataset, which is slightly (4.9%) lower than the maximum accuracy achieved with the AlexNet recognition scheme. However, the low accuracy on the PolyU dataset achieved by the proposed recognition scheme is much better than that of existing schemes. Hence, the proposed recognition scheme is capable of performing with great accuracy on palm vein images.

### 4.3. Area under Curve

**Figure 11:**
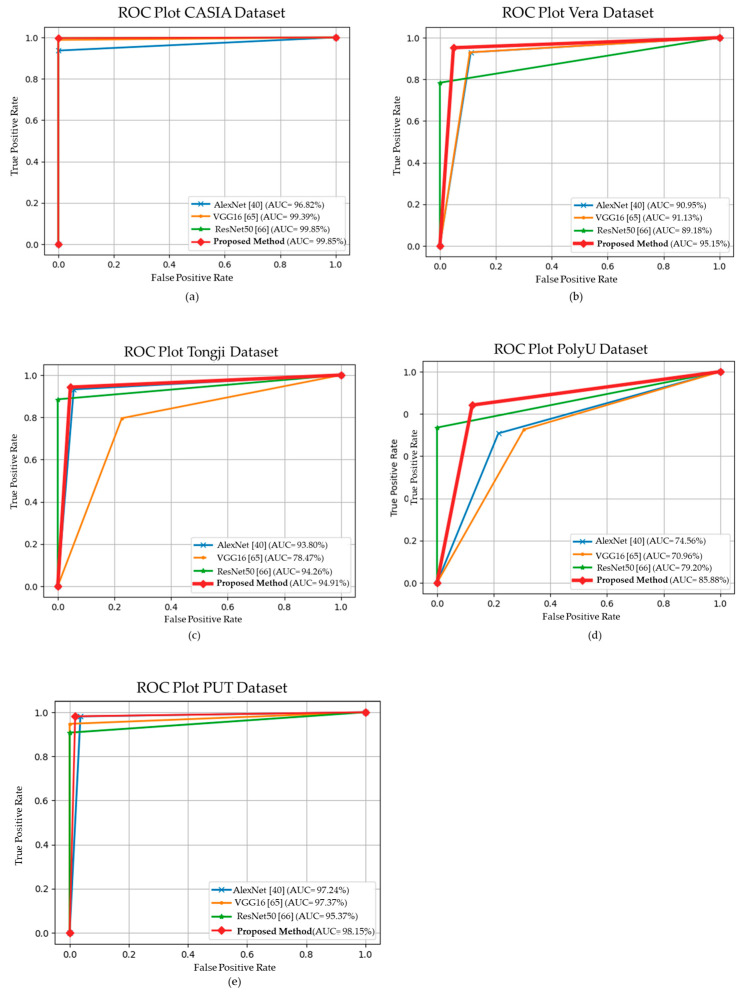
The AUC results of the transfer-learning-based methods on (**a**) CASIA, (**b**) Vera, (**c**) Tongji, (**d**) PolyU and (**e**) PUT dataset [40,65,66].

The proposed recognition scheme DWT-HOG VeinCNNalso results in an AUC as great as the accuracy on all datasets. The maximum AUC is achieved on the CASIA and PUT datasets with values of 99.85% and 98.15%, while the minimum AUC is attained on the PolyU dataset with an AUC of 85.88%. This value is 4.9% lower than the result attained with AlexNet, similar to results attained for accuracy as shown in Figure 11. This condition shows that the proposed scheme has adequate consistency. The results show that our proposed model attained the highest AUC of 95.3% on the Vera dataset.

### 4.4. EER

The EER generated by the proposed recognition scheme hybrid DWT-HOG VeinCNN is varied for all datasets, as shown in Table 8. The best EER value, 0.0083, is achieved by the proposed scheme on the CASIA dataset, so this scheme can avoid image misreading. The highest EER gained by this scheme, 0.0083, is 0.5% different from that achieved by AlexNet. Even so, the EER value generated by the proposed hybrid DWT-HOG VeinCNN recognition scheme is more consistent than others, including AlexNet, VGG16, and ResNet50. In fact, the average EER gained by the proposed scheme is 0.0592, 0.02% higher than that of AlexNet. The EER generated by the proposed scheme on the Tongji dataset is in line with that of the AlexNet recognition scheme, which is opposed to VGG16 and ResNet50 recognition schemes. Hence, the proposed recognition scheme hybrid DWT-HOG VeinCNN has the potential to avoid palm vein misreading.

## 5. Conclusions

This paper proposed a new scheme of CNN to recognize palm veins based on images. The proposed method, called VeinCNN, combines the features of DWT and HOG in order to robustly distinguish vein features.

VeinCNN starts from the fact that the acquisition process can be randomly affected by noise in indistinct images of veins. The recognition process can be affected by noisy images. Utilizing the wavelet transformation method allowed for the filtering of interference in venous images. After this transformation, the image is sharper and better prepared for the extraction of gradient features using HOG feature extraction. HOG features can acquire information properties that are more noise resistant and representative. The palm vein image has the potential to be an object to be automatically confirmed by a noise-resistant feature extraction method and modified CNN network.

The results show that wavelet transformation generally yields inferior results to HOG feature extraction. The best outcome was obtained when VeinCNN was hybridized with wavelet and HOG. Some datasets had an improvement in accuracy and AUC, but nearly all datasets had a decline in EER. The best result was obtained on the CASIA dataset with 99.85% accuracy, 99.80% AUC, and a 0.0083 EER value. Moreover, a total of 388,546 parameters used on the VeinCNN hybrid DWT and HOG method can maintain the results for accuracy, AUC, and EER.

According to the results, the proposed DWT-HOG VeinCNN method is a promising method compared with other recognition methods in transfer learning to obtain satisfactory palm vein recognition.

## Figures and Tables

**Table 1:** Comparison with state-of-the-art models.

Authors	Dataset	Feature Extractor	Method
Wang et al. (2018) [47]	PolyU	-	VGG-16
Qin et al. (2019) [43]	CASIA PolyU	-	DBN
Wulandari et al. (2019) [55]	PUT	DWT	CNN
Chantaf et al. (2020) [44]	Nonpublic	-	CNN
Kuang et al. (2020) [56]	CASIA PolyU	HOG	CNN
Chen et al. (2021) [46]	CASIA PUT	Gabor	CNN
Wu et al. (2023) [31]	Nonpublic CASIA Tongji PolyU	SDSPCA-NPE	Distance Feature Matching
Proposed	CASIA Vera Tongji PolyU PUT	Hybrid DWT and HOG	VeinCNN

**Table 2:** Summary of palm vein datasets.

No.	Dataset	Total Volunteers	Total Images	Number of Session	Image Size	Format	Official ROI
1.	CASIA	200	2400	2	768×576	jpg	Not Available
2.	Vera	220	2200	2	480×576	png	Available
3.	Tongji	600	12,000	2	800×600	tiff	Available
4.	PolyU	500	24,000	2	352×288	jpg	Available
5.	PUT	100	1200	3	768×1024	bmp	Not Available

**Table 3:** The procedure of the proposed hybrid DWT and HOG feature extraction.

Proposed Hybrid Feature Extractor DWT and HOG Procedure	
1:	$X_{m,n}\leftarrow Palm\;Vein\;Image\;(A_{m,n})$
2:	$\left({CA}_{\frac{m}{2},\frac{n}{2}},{CH}_{\frac{m}{2},\frac{n}{2}},{CV}_{\frac{m}{2},\frac{n}{2}},{CD}_{\frac{m}{2},\frac{n}{2}}\right)\leftarrow Haar\;Wavelet\;Transform\;(X_{m,n})$
3:	$Y_{m,n}\leftarrow Palm\;Vein\;Image\;({CA}_{\frac{m}{2},\frac{n}{2}})$
4:	$\left(G_{x},G_{y}\right)\leftarrow Gradient(Y_{m,n})$
5:	${Gradient}_{Magnitude}\leftarrow Magnitude\left(G_{x},G_{y}\right)$
6:	${Gradient}_{Direction}\leftarrow Direction\left(G_{x},G_{y}\right)$
7:	${HOG}_{Feature}\leftarrow Histogram\;Normalization\left({Gradient}_{Magnitude},{Gradient}_{Direction}\right)$
8:	${HOG}_{image}\leftarrow {HOG}_{Feature}$

**Table 4:** Summary of the VeinCNN network structure.

Layer	Type	Activation Function	Output Shape	Kernel Size	Number of Filters
0	Input	-	128 × 128	-	-
1	2D conv	ReLU	126 × 126	3	32
2	2D max pooling	ReLU	63 × 63	2	32
3	2D conv	ReLU	61 × 61	3	64
4	2D max pooling	ReLU	30 × 30	2	64
5	2D conv	ReLU	28 × 28	3	64
6	2D max pooling	ReLU	14 × 14	2	64
7	2D conv	ReLU	12 × 12	3	64
8	2D max pooling	ReLU	6 × 6	2	64
9	Flattened	-	2304	-	-
10	Dense	ReLU	128	-	-
11	Dense	Sigmoid	2	-	-

**Table 5:** The result of accuracy, AUC, and EER comparison methods.

Dataset	Feature Extractor	Accuracy (%)	AUC (%)	EER
CASIA	Raw data	99.69	99.60	0.0167
	DWT	99.85	98.80	0.0250
	HOG	99.85	99.80	0.0083
	Hybrid DWT and HOG (proposed)	99.85	99.80	0.0083
Vera	Raw data	84.14	84.10	0.1273
	DWT	81.16	81.20	0.2000
	HOG	94.78	94.60	0.0636
	Hybrid DWT and HOG (proposed)	95.57	95.10	0.0545
Tongji	Raw data	90.37	90.40	0.1383
	DWT	92.04	92.00	0.0866
	HOG	94.81	94.40	0.0750
	Hybrid DWT and HOG (proposed)	94.91	94.90	0.0650
PolyU	Raw data	59.44	59.40	0.3000
	DWT	58.33	60.20	0.2566
	HOG	82.59	82.60	0.2066
	Hybrid DWT and HOG (proposed)	85.88	85.90	0.1467
PUT	Raw data	93.52	93.50	0.1000
	DWT	74.07	81.30	0.1330
	HOG	97.22	97.20	0.0333
	Hybrid DWT and HOG (proposed)	98.12	98.10	0.0167

**Table 6:** Summary of accuracy, AUC, and EER for each dataset using the proposed method.

Dataset	Accuracy (%)	AUC (%)	EER
CASIA	99.85	99.80	0.0083
Vera	95.57	95.10	0.0545
Tongji	94.91	94.90	0.0650
PolyU	85.88	85.90	0.1467
PUT	98.12	98.10	0.0167

**Table 7:** The accuracy results of transfer-learning-based methods on datasets.

Methods	Accuracy on Dataset (%)				
	CASIA	Vera	Tongji	PolyU	PUT
AlexNet [38]	96.60	90.86	93.80	73.98	97.22
VGG16 [67]	99.38	91.04	78.43	70.83	97.22
ResNet50 [68]	99.69	86.38	88.43	79.17	94.44
Proposed hybrid DWT-HOG VeinCNN	99.85	95.52	94.91	85.97	98.15

**Table 8:** The EER results of transfer-learning-based methods on datasets.

Recognition Scheme	EER on Dataset				
	CASIA	Vera	Tongji	PolyU	PUT
AlexNet [38]	0.0679	0.0672	0.0685	0.3370	0.0185
VGG16 [67]	0.0123	0.0672	0.1963	0.2528	0.0555
ResNet50 [68]	0.0083	0.0560	0.1167	0.1519	0.0185
Proposed Hybrid DWT-HOG VeinCNN	0.0083	0.0545	0.0630	0.1460	0.0167

## Data Availability

This manuscript uses image dataset which are from Chinese Academy of Sciences Institute of Automation (CASIA) [50], Hong Kong Polytechnic University (PolyU) [51], PUT [52], Tongji [53], and University of Applied Sciences Western Switzerland and the Idiap Research Institute [54].
